# Preservation of biomolecules in breast cancer tissue by a formalin-free histology system

**DOI:** 10.1186/1472-6890-8-1

**Published:** 2008-01-29

**Authors:** Mehdi Nassiri, Sharon Ramos, Hajir Zohourian, Vladimir Vincek, Azorides R Morales, Mehrdad Nadji

**Affiliations:** 1 Department of Pathology, University of Miami Miller School of Medicine, Miami, Florida, USA; 2 Department of Pathology, University of Florida, Gainesville, Florida, USA

## Abstract

**Background:**

The potential problems associated with the use of formalin in histology, such as health hazards, degradation of RNA and cross-linking of proteins are well recognized. We describe the utilization of a formalin-free fixation and processing system for tissue detection of two important biopredictors in breast cancer – estrogen receptor and HER2 – at the RNA and protein levels.

**Methods:**

Parallel sections of 62 cases of breast cancer were fixed in an alcohol-based molecular fixative and in formalin. Molecular fixative samples were processed by a novel formalin-free microwave-assisted processing system that preserves DNA, RNA and proteins. Formalin-fixed samples were processed using the conventional method. Estrogen receptor was assessed by immunohistochemistry and real-time PCR. HER2 was assessed by immunohistochemistry, FISH, CISH and real-time PCR.

**Results:**

The immunohistochemical reaction for estrogen receptor was similar in molecular- and formalin-fixed samples (Spearman Rank R = 0.83, p < 0.05). Also HER2 result was similar to that of formalin-fixed counterparts after elimination of antigen retrieval step (Spearman Rank R = 0.84, p < 0.05). The result of HER2 amplification by FISH and CISH was identical in the molecular fixative and formalin-fixed samples; although a shorter digestion step was required when using the former fixative. Real-time PCR for both estrogen receptor and HER2 were successful in all of the molecular fixative specimens.

**Conclusion:**

The formalin-free tissue fixation and processing system is a practical platform for evaluation of biomolecular markers in breast cancer and it allows reliable DNA and RNA and protein studies.

## Background

Formaldehyde-fixed, paraffin-embedded tissue (FFPE) is the product of a century old histopathology practice [1]. However, tissue processed by this system has limited application beyond routine histology and immunohistochemistry. For example, most of the current clinical molecular tests require fresh tissue. Fresh or fresh-frozen tissue specimens, on the other hand, are of limited value for the assessment of histomorphology, and are impractical for long-term retrospective studies due to their inherent logistical and storage problems [2,3].

It is not surprising, therefore, that alternative formalin-free tissue handling methodologies have been introduced in recent years. We have previously reported our experience with a new, simple and practical, yet standardized, tissue fixation and processing method that preserves histomorphology and protects macromolecules at ambient temperature [4-9]. This method is easily applicable to both clinical and research settings. It includes standard tissue sectioning, fixation by an alcohol-based fixative, and processing in an automated formalin-free microwave-based tissue processor. Here we report results from studies of breast cancer processed with this new molecular-friendly platform and compare it with conventional tissue processing.

## Methods

### Tissue Samples

Paired tissue section of similar dimensions, 1.5 × 1.5 × 0.2 cm were taken from 62 surgically excised breast cancer specimens and fixed in UMFIX (Universal Molecular Fixative, marketed as Tissue-Tek^® ^Xpress™ Molecular Fixative, Sakura Finetek, Torrance, CA) and 10% neutral buffered formalin. Molecular fixative is composed of methanol and polyethylene glycol at predetermined ratio (US patent # 7,138,226). All samples were immersed in fixative within 30 minutes of surgery. Immersion time was similar for both fixatives for each specimen (less than 24 hours for 32 cases, 24–48 hours for 18 cases, 48–72 hours for 12 cases). Formalin-fixed samples were then processed using a conventional method (VIP, Sakura Finetek, Torrance, CA). Samples fixed in molecular fixative were processed by a recently described automated microwave-based rapid tissue-processing instrument (Tissue-Tek^® ^Xpress™, Sakura Finetek, Torrance, CA). H&E-stained slides were reviewed to confirm the presence of tumor and the non-neoplastic mammary epithelium. All studies were approved by the University of Miami Institutional Review Board.

### Immunohistochemistry

Three-micron sections mounted on charged slides were stained for estrogen receptor (ER) using the ER pharmDx Kit™ (Dako, Carpinteria, CA) following the kit's protocol. Adjacent sections of each case were also stained with a monoclonal antibody to ER (clone 1D5) following a previously published protocol [10] that includes an antigen retrieval step and L-SAB detection system (Dako). The number of positive nuclei and the intensity of reactions were evaluated and graded in tumor cells according to the respective protocols. When present, the reaction in adjacent non-neoplastic mammary epithelium served as the internal control. The intensity and pattern of reactions were compared in tissue samples fixed by the two methods.

The HercepTest kit (Dako, Carpinteria, CA) was used for HER2 immunohistochemistry. The kit's protocol was strictly followed to for both types of samples. In addition, one section from the UMFIX-exposed tumors was stained without the antigen retrieval step. The HER2 reactions were scored according to ASCO/CAP recommendations [11]. A positive result for HER2 was considered when IHC staining of 3+ (uniform, intense membrane staining of >30% of invasive tumor cells) was present.

### HER2-FISH and HER2-CISH

Chromogenic in situ hybridization for HER2 gene amplification was performed using Zymed (South San Francisco, CA) probe and reagents. For HER2-FISH, reagents and probes were from Dako. A duplicate slide of all UMFIX tumors was used to repeat FISH and CISH procedures with reduced enzyme digestion times (from 25 minutes to 7 minutes for FISH and from 20 to 5 minutes for CISH). The reduction in enzyme digestion time was necessary to prevent the overdigestion of UMFIX tissue sections resulting in their loss during the wash steps. The interpretation of results and scoring was carried out following the manufacturers' guidelines. A fluorescent in situ hybridization (FISH) ratio (HER2 gene signals to chromosome 17 signals) of more than 2.2 was considered positive or amplified; a FISH ratio of less than 1.8 was considered negative or not amplified.

To evaluate the IHC and CISH staining, random selections of slides were evaluated simultaneously on a multi-headed microscope by three pathologists and a consensus score was reported for each slide. FISH slides were scored by one pathologist. All slides had been stripped of any identifier referring to fixation or processing method.

### RNA and DNA Studies

RNA was extracted from 50-micron thick sections of the paraffin-embedded tissue. Extraction was performed by addition of Trizol reagent (Invitrogen, Carlsbad, CA) and subsequent homogenization using a Tissue Tearor (Biospec Products Inc., Bartlesville, OK). The RNA from homogenized tissue was extracted using aqueous phase separation by chloroform followed by isopropyl precipitation on ice. The RNA pellet was further purified on Qiagen (Valencia, CA) RNAeasy column and treated with DNase. A standard 1% agarose gel under denaturing conditions with ethidium bromide was used to assess the integrity of RNA. In addition, RNA was evaluated on an Agilent Technologies Bioanalyzer 2100 using RNA 6000 Nano Chips (Lindenhurst, NY) to determine the RNA integrity and the ratio of ribosomal RNA. Quantitation was performed with ND-1000 Spectrophotometer (NanoDrop Technologies Wilmington, DE).

DNA was isolated from the organic phase of the Trizol tissue homogenate using Gentra (Minneapolis, MN) Puregene Kit and treated with RNase. The quantity of the extracted RNA and DNA was determined by spectrophotometery (NanoDrop Technologies Wilmington, DE)). Two micrograms of cleaned RNA was reverse transcribed to cDNA using Invitrogen (Carlsbad, CA) Cloned AMV cDNA synthesis kit and random hexamers.

PCR was performed using primers for estrogen receptor (ESR1 transcript ENST00000206249, 114 bp spanning exons 3 and 4, Sense 5'-GTGGGATACGAAAAGACCGAAGA, Antisense 5'-GGTTGGCAGCTCTCATGTCTC) and HER2 (ERBB2 transcript ENST00000269571, 113 bp spanning exons 2 and 3, sense 5'-GGGAAACCTGGAACTCACCTAC, Antisense 5'-GGACCTGCCTCACTTGGTTG). For real-time PCR 0.5 μg of cDNA or RNase-treated DNA was used as the template, utilizing Qiagen Quantitect Sybrgreen Mastermix on a Bio-Rad I-cycler (Hercules, CA). Serial dilutions of a known quantity of template for each gene were used to measure the copy numbers. To create the standard dilution series templates, ER and HER2 amplicons were cloned from pooled samples of known ER- and HER2-positive breast cancer samples using Invitrogen TOPO TA cloning kit. Only copy numbers above "1" were considered reliable values for further data analysis. Real-time PCR of Cyclophilin A (PPIA, ENST00000244636, Biosource Camarillo, CA) and 7SL RNA (RN7SL1, NR_002715.1 GI:84871994, sense ACCACCAGGTTGCCTAAGGA, Antisense 5'-CACGGGAGTTTTGACCTGCT) was performed as control. Conditions for all real-time PCR reactions were an initial Taq activation at 95°C for 20 minutes followed by 40 cycles at 95°C for 15 seconds, and 60°C for 1 minute.

Twenty samples were studied using β-actin (ENST00000331789) primers to amplify transcripts ranging from 131 to 705 bp as described before [12] with following primer sets: sense 5'-CCACACTGTGCCCATCTACG, antisense-1 131 bp CCGTGGTGGTGAAGCTGTAG, antisense-2 291 bp CAGCGGAACCGCTCATTGCCAATGG, antisense-3 402 bp TACAGGTCTTTGCGGATGTCCA, antisense-4 502 bp GATCTTCATTGTGCTGGGTGCC, antisense-5 601 bp CTGCTTGCTGATCCACATCTG, antisense-6 705 bp CTGCGCAAGTTAGGTTTTGTC. Amplified products were run on an Agilent Technologies Bioanalyzer 2100 using DNA 1000 LabChip kit (Lindenhurst, NY). PCR was performed for a 450 bp fragment of Glyceraldehyde-3-phosphate dehydrogenase DNA (GAPDH, ENSG00000111640) with commercial primers from Clonetech (Palo Alto, CA) using 0.5 μg of RNase-treated isolated DNA and Qiagen TaqPCR Mastermix (Qiagen, Valencia, CA). The conditions for DNA PCR were: 95°C, 15 minutes; 35 cycles at 94°C, 45 seconds; 60°C, 45 seconds; 72°C, 2 minutes.

Samples were stripped of any identifier referring to fixation or processing method during all experimental steps. Statistical analysis was performed with the aid of Statistica software (StatSoft, Tulsa, OK).

## Results

The presence of invasive mammary carcinoma was confirmed on H & E slides of all 62 samples. In 58 cases there was normal and/or hyperplasic breast epithelium present adjacent to tumor.

**Figure 1:**
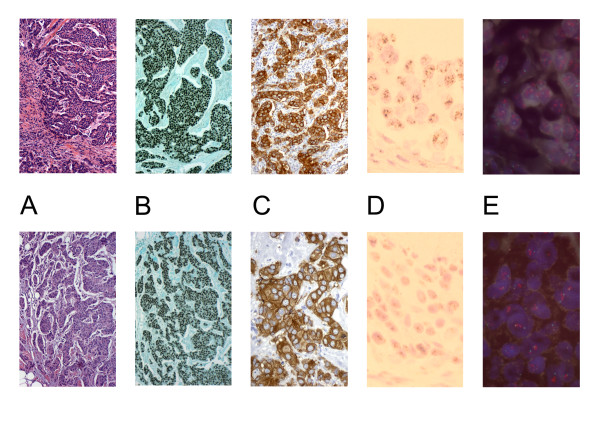
Representative histology (A) and immunohistochemistry for ER (B) and HER2 (40×) (C), and chromogenic (60×) (D) and fluorescent (E) in situ hybridization for HER2 (100×). (Top = UMFIX, Bottom = Formalin).

**Figure 2:**
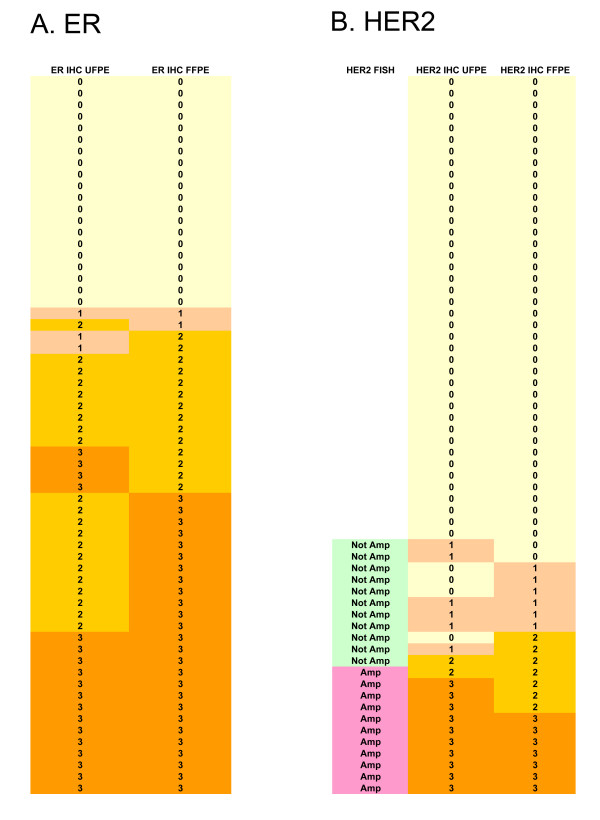
Heat map depiction of immunohistochemistry consensus score for ER (A) and HER2 (B) for UFPE and FFPE samples. Data were sorted based on FFPE score results. Category (top) describes the result of ER IHC (0-3+), FISH for both UMFIX and Formalin samples (Not Amp: not amplified, Amp: amplified), HER 2 IHC (0-3+). HER2 FISH result were the same in UFPE and FFPE sections, results only are shown for HER2 IHC 1-3+ cases. Each row represents one case.

Overall 42 (68%) of formalin- and UMFIX-exposed paraffin-embedded (UFPE) tumors were positive for ER. The ER reaction was diffuse in distribution and uniform in intensity in more than 90% of the nuclei of all positive tumors (Figures 1). Conversely, in adjacent benign epithelia, strongly positive, weakly positive and negative nuclei were randomly distributed. The number and the intensity of positive nuclei were similar in ER-pharmDX kit- and ER-1D5-stained tumors (Spearman Rank R = 0.88 p < 0.05). Similarly, the ER reaction in tumor cells and in non-neoplastic epithelium in formalin-fixed tissue and molecular fixative-exposed specimens was exactly the same when a dichotomized score (positive/negative) was used. Using a 0-3+ score scheme there was a good correlation between UFPE and FFPE samples (Spearman Rank R = 0.83 p < 0.05) and there was no significant difference (Wilcoxon matched pair test). There were no false-negative results (Figure 2A).

The overall staining intensity of HercepTest in UMFIX breast cancers was greater than formalin-fixed tumors. When the antigen retrieval step for UMFIX samples was omitted, however, the scoring of HercepTest in UMFIX and formalin samples became similar (Spearman Rank R = 0.84 p < 0.05, Wilcoxon test p = not significant).

Regarding the predictive potential of ER and HER2 immunohistochemistry, no case was misclassified in UFPE tissue (ER positive, HER2 positive or 3+). All ER positive cases in FFPE samples were also positive in UFPE tissue. Although more case were HER2 positive (HER2 3+) in UFPE tissue, these cases also showed amplification by FISH. Furthermore, in UFPE samples only two cases were considered indeterminate for HER2 by immunohistochemistry (HER2 score of 2+) compared to seven FFPE samples (Figure 2B). We have not evaluated the suitability of HercepTest performed on tissues fixed in molecular fixative for predicting response to Herceptin. There were no discrepancies of HER2-FISH and HER2-CISH results between UMFIX and formalin samples. There were no equivocal cases by FISH in our series (HER2/Chromosome 17 ratio of 1.8–2.2).

**Figure 3:**
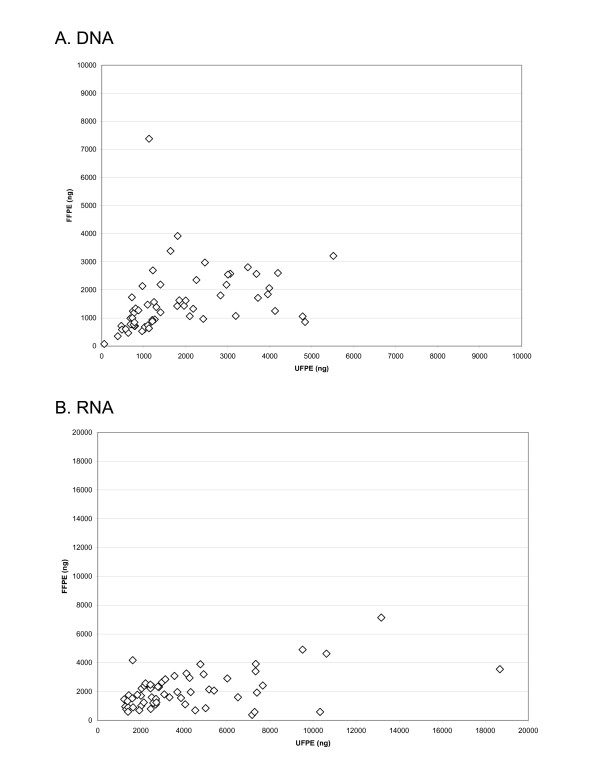
Scatter plot of the yield (in nanogram) of DNA (A) and RNA (B) from 50 micron thick sections of UFPE – and FFPE breast cancer.

**Figure 4:**
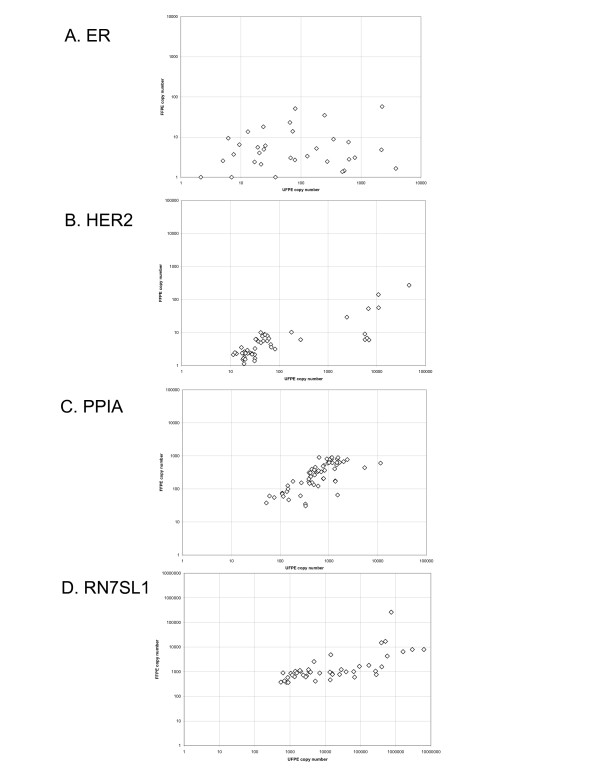
Scatter plot (logarithmic scale) of transcript copy number data for ER (A), HER2 (B), Cyclophilin A (PPIA) (C) and RN7SL1 (D).

While the DNA yield was similar in formalin and UMFIX samples (Figure 3A), the RNA yield was significantly higher in the latter (p < 0.05 t-test, Figure 3B). Using the same amount of RNA template (2 μg), the cDNA yield was similar between two groups. Real-time PCR for HER2- and ER-RNA showed at least a ten fold difference in transcript copy number between UMFIX and formalin samples (Figure 4). Two control transcripts, PPIA and RN7sL had different dynamic ranges. PPIA was, on average, twofold higher in UMFIX samples. However, RN7SL1 copies were 50 times higher in UMFIX samples. These results indicate greater reverse-transcription efficiency and amplification in the UMFIX samples.

**Figure 5:**
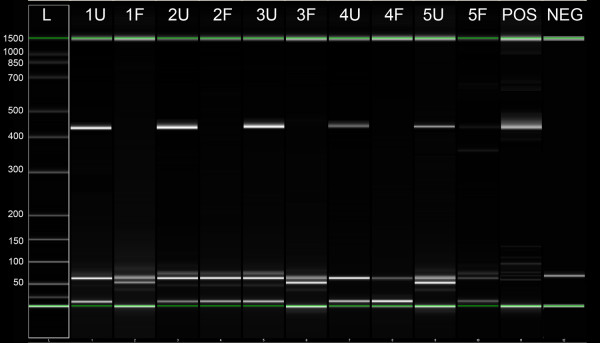
Agilent DNA 1000 chip result of 450-base pair GAPDH DNA PCR product in five paired breast cancer samples. L = ladder, numbers in base-pairs, U = UFPE, F = FFPE, POS = positive control NEG = no template.

**Figure 6:**
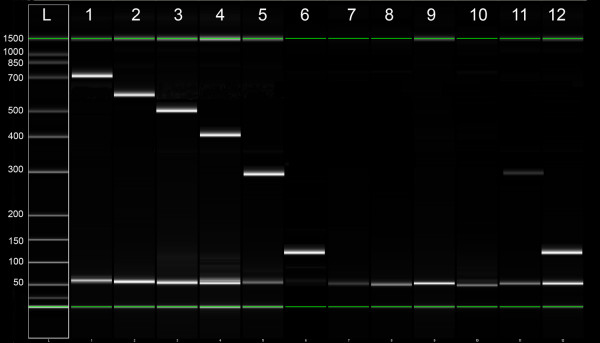
Representative result of PCR for β-actin RNA from a paired set of UFPE and FFPE breast cancer tissue. PCR products analyzed using Agilent DNA 1000 chip. L = ladder, numbers in base-pairs, Lane 1–6 = UFPE, 7–12 = FFPE, 1 and 7 = 705 bp, 2 and 8 = 601 bp, 3 and 9 = 502 bp, 4 and 10 = 402 bp, 5 and 11 = 291 bp, 6 and 12 = 131 bp.

Larger size amplicons could be reliably amplified in UMFIX samples but not from formalin-fixed tissues. For example, a 450-bp segment of GAPDH DNA could be easily detected in all UMFIX samples whereas it was rarely amplified from formalin-fixed tissue (Figure 5). We studied twenty samples with PCR for β-actin to evaluate the length of a transcript that can be amplified. Only products up to 291 bp could be seen in formalin fixed samples. In contrast, all UMFIX samples showed the 705 bp product (Figure 6).

## Discussion

The current formalin-based methods of fixation and processing of tissue for histopathological evaluation hinder the reliable analysis of macromolecules. For example, of many available ER antibodies, only a few reliably react with formalin-fixed paraffin-embedded tissue antigens [10]. However, several other antibodies cannot be optimized for use in formalin fixed tissue as the cross-linking of the target protein leads to masking of the antigenic sites [13-15]. Similarly, DNA and RNA are not well preserved when formalin is used as a fixative. It has been shown that formaldehyde fixation results in nucleic acid fragmentation [16]. Thus nucleic acid studies conducted on conventionally processed tissue have been limited to amplification of small size amplicons. Nucleic acid segments larger than 200-bp cannot be reproducibly amplified from formalin-fixed material [17]. Formalin fixation also introduces conformational and chemical changes in DNA that lead to infidelity of DNA replication by polymerases. Chemical modification of RNA by formalin seriously compromises the reliability of amplification methods [18,19].

Development of quantitative molecular assays using FFPE tissue has been difficult. Beside inherent problems in creating standards in tissue-based assays, formalin-fixed tissues require different methodological approaches compared to fresh samples. For example, Specht et al studied FFPE samples and fresh tissue after microdissection and compared different extraction RNA methods [20]. They show, by modification of digestion, extraction and amplification methods it is possible to achieve results similar to fresh samples. Also Gjerdrum et al describe use of microdissected tumor cells from FFPE breast cancer tissue for HER2-RNA and DNA study with success rate of 97% for DNA and 94% for RNA. However they used gene-specific antisense primers during the reverse transcription phase rather than random hexamers, and amplified very short sequences of 72 base-pairs for HER2. Despite their promising result these authors suggest that HER2 gene expression data from FFPE tissue studies should be used with caution, especially in the clinical setting [21]. This may be due to significant differences in the replicate results of mRNA levels within individual patients. They attribute this irreproducibility to both tumor heterogeneity and technical inaccuracies. Therefore, despite progress in the technical aspects of studying FFPE samples, they have not yet gained widespread acceptance due to the lack of reproducibility.

There have been many attempts in recent years to introduce alternative methods of tissue fixation and processing [22-24]. Most have impractical aspects for routine laboratory use; e.g. the requirement for fixation and/or processing at or below -4°C, or the lack of potential for high throughput automation. With few exceptions, these methods and reagents have not been evaluated or validated for molecular studies [25,26].

We recently developed a continuous flow, high throughput formalin-free tissue processing system (CRTP) and UMFIX, an alcohol-based molecular-friendly fixative. Paraffin blocks of tissues fixed and processed by this system yield high quality histomorphology along with preservation of intact high molecular weight nucleic acids and proteins in the paraffin-embedded blocks [7,8].

The current study demonstrates that this platform is not only reliable for immunohistochemistry of ER and HER2 in breast cancer, but also for DNA and RNA studies. We used the same methods for extraction of DNA and RNA from fresh tissue for UMFIX/CRTP samples and were able to amplify large amplicons in all of them. Furthermore, application of the new methods required less time compared to formalin-fixed material. While conventional formalin-fixed, overnight processed tissue samples have a considerable failure and reproducibility issues for DNA or RNA studies, we have yet to encounter a single specimen prepared with our method that its nucleic acids could not be used for downstream applications. Better preservation of DNA and RNA was evident in our samples by greater amplification efficiency. Immunohistochemical studies require many technical optimization steps. An often neglected pre-analytical step is the tissue fixation part, which includes time to fixation, fixative, and duration of fixation. Lack of strict criteria in tissue handling steps has direct effect on therapeutic decisions that are being made based on tissue markers expression [10,27]. Recent ASCO/CAP recommendation has addressed some of these steps [12]. For example, therapeutic decision is based on HER2 3+ positive results by IHC or evidence of amplification by FISH. Since performance of FISH as a primary test is prohibitively expensive, many institutions use a two-tier system of performing IHC as the first step and FISH as the second step. Cases with Her2-IHC score of 2+ are considered equivocal and are not treatment-candidate. These cases need to be confirmed by FISH and must show amplification in order to receive Herceptin. In our study, 7 formalin-fixed samples had 2+ IHC score; three of these cases had 3+ score in parallel UMFIX section (all showed amplification by FISH) and two had 2+ score (one with amplification by FISH, Figure 2B). Therefore UFPE samples had lower number of equivocal HER2 IHC score compared to FFPE samples. Since all of the 3+ IHC positive UFPE cases were also positive by FISH, there was no false positive result that might affect therapeutic decision based on HER2 IHC score of UFPE tissue. On the other hand, fewer cases with equivocal IHC scores in UFPE blocks means decrease in laboratory workload to perform FISH studies to validate these results. This might decrease the financial burden of performing more expensive FISH assay to confirm the equivocal IHC results. Clinical application of HercepTest performed on tissues fixed in molecular fixative need to be further studied in a larger series of cases and correlated with the response to Herceptin. However, this method seems to be associated with fewer fixative-related artifacts. With the new methods, the pre-analytical quality of tissue samples are assured and standardized before they are used for expensive molecular testing – an important consideration given the growing importance of assaying archival specimens for the suitability of new therapy. The current lack of standards in tissue handling, as well as complex chemical reactions that take place during routine formalin-based tissue processing, make it unlikely that the final product can serve as a reliable source for quantitative molecular diagnostics.

## Conclusion

Medical practice is increasingly dependent upon accurate molecular diagnostics. The present study shows that it is feasible to simultaneously evaluate histomorphology and perform reliable molecular studies on fixed and processed human breast cancer tissue. The DNA, RNA, and protein epitopes are all preserved, intact in paraffin-embedded UMFIX/CRTP tissue, enabling current, routine, and future, as yet to be imagined molecular studies.

## Abbreviations

bp = base pair, IHC = immunohistochemistry, UFPE = UMFIX-exposed paraffin embedded, FFPE = Formalin-fixed paraffin embedded

## Competing interests

The authors have been granted one (M Nassiri, V Vincek, M Nadji) or more (AR Morales) patents for their inventions in histology. The University of Miami licensed these inventions which are the basis of UMFIX fixative (Tissue-Tek^® ^Xpress™ Molecular Fixative), and Tissue-Tek^® ^Xpress™ tissue processor to Sakura Finetek, Inc. The authors have received royalties and research support from Sakura Finetek.

## Authors' contributions

This study was planned by MNassiri, MNadji, VV and ARM. Samples were collected and evaluated by MNassiri, MNadji and ARM. Experiments were performed by MNassiri, HZ and SR. MNassiri analyzed the data. Manuscript was prepared by MNassiri and MNadji. All authors have read and approved the final version of the manuscript. The authors are grateful to Dr Christian Wunsch for his comments and criticism.

## Pre-publication history

The pre-publication history for this paper can be accessed here:

http://www.biomedcentral.com/1472-6890/8/1/prepub

## References

[B1] Eltoum I Fredenburgh J Myers RB Grizzle WE Introduction to the theory and practice of fixation of tissues J Histotech 2001 24 173 190

[B2] Emmert-Buck MR Strausberg RL Krizman DB Bonaldo MF Bonner RF Bostwick DG Brown MR Buetow KH Chuaqui RF Cole KA Duray PH Englert CR Gillespie JW Greenhut S Grouse L Hillier LW Katz KS Klausner RD Kuznetzov V Lash AE Lennon G Linehan WM Liotta LA Marra MA Munson PJ Ornstein DK Prabhu VV Prang C Schuler GD Soares MB Tolstoshev CM Vocke CD Waterston RH Molecular profiling of clinical tissues specimens: feasibility and applications J Mol Diagn 2000 2 60 6 11272889 10.1016/s1525-1578(10)60617-4 PMC1906897

[B3] Nassiri M Nadji M Tissue detection of biomolecular predictors in breast cancer Expert Rev Anticancer Ther 2006 6 1225 32 10.1586/14737140.6.8.1225 16925488

[B4] Cleary TJ Morales AR Nadji M Nassiri M Vincek V Antimicrobial activity of UMFix tissue fixative J Clin Pathol 2005 58 22 5 10.1136/jcp.2004.021618 15623477 PMC1770532

[B5] Morales AR Essenfeld H Essenfeld E Duboue MC Vincek V Nadji M Continuous specimen-flow, high-throughput, 1-hour tissue processing: a system for rapid diagnostic tissue preparation Arch Pathol Lab Med 2002 126 583 590 11958665 10.5858/2002-126-0583-CSFHTH

[B6] Morales AR Nassiri M Kanhoush R Vincek V Nadji M Experience with an automated microwave-assisted rapid tissue processing method validation of histologic quality and impact on the timeliness of diagnostic surgical pathology Am J Clin Pathol 2004 121 528 36 10.1309/ACK8-AHV0-1T47-QR53 15080304

[B7] Nadji M Nassiri M Vincek V Kanhoush R Morales AR Immunohistochemistry of tissue prepared by a molecular-friendly fixation and processing system Appl Immunohistochem Mol Morphol 2005 13 277 82 10.1097/01.pai.0000146544.51771.79 16082256

[B8] Vincek V Nassiri M Nadji M Morales AR A tissue fixative that protects macromolecules (DNA, RNA, and protein) and histomorphology in clinical samples Lab Invest 2003 83 1 9 10.1097/01.LAB.0000090154.55436.D1 14563944

[B9] Nassiri M Gugic D Olczyk J Ramos S Vincek V Preservation of Skin DNA for Oligonucleotide Array CGH Studies, A Feasibility Study Arch Dermatol Res 2007 299 353 7 10.1007/s00403-007-0773-6 17665208 PMC1950585

[B10] Nadji M Gomez-Fernandez C Ganjei-Azar P Morales AR Immunohistochemistry of estrogen and progesterone receptors reconsidered: experience with 5,993 breast cancers Am J Clin Pathol 2005 123 21 7 10.1309/4WV7-9N2G-HJ3X-1841 15762276

[B11] Chen J Byrne GE Jr Lossos IS Optimization of RNA extraction from formalin-fixed, paraffin-embedded lymphoid tissues Diagn Mol Pathol 2007 16 61 72 10.1097/PDM.0b013e31802f0804 17525674

[B12] Wolff AC Hammond ME Schwartz JN Hagerty KL Allred DC Cote RJ Dowsett M Fitzgibbons PL Hanna WM Langer A McShane LM Paik S Pegram MD Perez EA Press MF Rhodes A Sturgeon C Taube SE Tubbs R Vance GH van de Vijver M Wheeler TM Hayes DF American Society of Clinical Oncology; College of American Pathologists American Society of Clinical Oncology/College of American Pathologists guideline recommendations for human epidermal growth factor receptor 2 testing in breast cancer Arch Pathol Lab Med 2007 131 18 43 19548375 10.5858/2007-131-18-ASOCCO

[B13] Nadji M Morales AR Immunoperoxidase: Part I. Technique and its pitfalls Lab Med 1983 14 767 771

[B14] Puchtler H Meloan SN On the chemistry of formaldehyde fixation and its effects on immunological reactions Histochem 1985 82 201 204 10.1007/BF00501395 3997553

[B15] Werner M Chott A Fabiano A Battifora H Effect of formalin fixation and processing on immunohistochemistry Am J Surg Pathol 2000 24 1016 1019 10.1097/00000478-200007000-00014 10895825

[B16] Feldman NY Reaction of nucleic acids and nucleoproteins with formaldehyde Prog Nucleic Acid Res Mol Biol 1973 13 1 49 4573489 10.1016/s0079-6603(08)60099-9

[B17] Lehmann U Kreipe H Real-time PCR analysis of DNA and RNA extracted from formalin-fixed and paraffin-embedded biopsies Methods 2001 25 409 418 10.1006/meth.2001.1263 11846610

[B18] Masuda N Ohnishi T Kawamoto S Monden M Okubo K Analysis of chemical modification of RNA from formalin-fixed samples and optimization of molecular biology application for such samples Nucl Acids Res 1999 27 4436 4443 10.1093/nar/27.22.4436 10536153 PMC148727

[B19] Rait VK Zhang Q Fabris D Mason JT O'Leary TJ Conversions of formaldehyde-modified 2-Deoxyadenosine 5'-Monophosphate in conditions modeling formalin-fixed tissue dehydration J Histochem Cytochem 2005 54 301 10 10.1369/jhc.5A6725.2005 16116034 PMC1783762

[B20] Specht K Richter T Muller U Walch A Werner M Hofler H Quantitative gene expression analysis in microdissected archival formalin-fixed and paraffin-embedded tumor tissue Am J Pathol 2001 158 419 428 11159180 10.1016/S0002-9440(10)63985-5 PMC1850313

[B21] Gjerdrum LM Sorensen BS Kjeldsen E Sorensen FB Nexo E Hamilton-Dutoit S Real-time quantitative PCR of microdissected paraffin-embedded breast carcinoma: an alternative method for HER-2/neu analysis J Mol Diag 2004 6 42 51 10.1016/S1525-1578(10)60490-4 PMC1867459 14736826

[B22] Gillespie JW Best CJ Bichsel VE Cole KA Greenhut SF Hewitt SM Ahram M Gathright YB Merino MJ Strausberg RL Epstein JI Hamilton SR Gannot G Baibakova GV Calvert VS Flaig MJ Chuaqui RF Herring JC Pfeifer J Petricoin EF Linehan WM Duray PH Bova GS Emmert-Buck MR Evaluation of non-formalin tissue fixation for molecular profiling studies Am J Pathol 2002 160 449 57 11839565 10.1016/S0002-9440(10)64864-X PMC1850633

[B23] Shibutani M Uneyama C Miyazaki K Toyoda K Hirose M Methacarn fixation: a novel tool for analysis of gene expressions in paraffin-embedded tissue specimens Lab Invest 2000 80 199 208 10701689 10.1038/labinvest.3780023

[B24] Warmington AR Wilkinson JM Riley CB Evaluation of ethanol-based fixatives as a substitute in a diagnostic clinical laboratories J Histotechnol 2000 23 299 308

[B25] Delfour C Roger P Bret C Berthe ML Rochaix P Kalfa N Raynaud P Bibeau F Maudelonde T Boulle N RCL2, a new fixative, preserves morphology and nucleic acid integrity in paraffin-embedded breast carcinoma and microdissected breast tumor cells J Mol Diag 2006 8 157 169 10.2353/jmoldx.2006.050105 PMC1867597 16645201

[B26] Uneyama C Shibutani M Masutomi N Takagi H Hirose M Methacarn fixation for genomic DNA analysis in microdissected, paraffin-embedded tissue specimen J Histochem Cytochem 2002 52 903 913 10.1177/002215540205000911 12185202

[B27] Rhodes A Jasani B Anderson E Dodson AR Balaton AJ Evaluation of HER-2/neu immunohistochemical assay sensitivity and scoring on formalin-fixed and paraffin-processed cell lines and breast tumors: a comparative study involving results from laboratories in 21 countries Am J Clin Pathol 2002 118 408 17 10.1309/97WN-W6UX-XJWT-02H2 12219783

